# EV-A71 vaccine licensure: a first step for multivalent enterovirus vaccine to control HFMD and other severe diseases

**DOI:** 10.1038/emi.2016.73

**Published:** 2016-07-20

**Authors:** Qunying Mao, Yiping Wang, Lianlian Bian, Miao Xu, Zhenglun Liang

**Affiliations:** 1 National Institutes for Food and Drug Control, Beijing 100050, China

**Keywords:** clinical manifestation, enterovirus 71 (EV-A71), foot and mouth disease (HFMD), hand, multivalent vaccine

## Abstract

Enteroviruses (EVs) are the most common viral agents in humans. Although most infections are mild or asymptomatic, there is a wide spectrum of clinical manifestations that may be caused by EV infections with varying degrees of severity. Among these viruses, EV-A71 and coxsackievirus (CV) CV-A16 from group A EVs attract the most attention because they are responsible for hand, foot and mouth disease (HFMD). Other EV-A viruses such as CV-A6 and CV-A10 were also reported to cause HFMD outbreaks in several countries or regions. Group B EVs such as CV-B3, CV-B5 and echovirus 30 were reported to be the main pathogens responsible for myocarditis and encephalitis epidemics and were also detected in HFMD patients. Vaccines are the best tools to control infectious diseases. In December 2015, China's Food and Drug Administration approved two inactivated EV-A71 vaccines for preventing severe HFMD.The CV-A16 vaccine and the EV-A71-CV-A16 bivalent vaccine showed substantial efficacy against HFMD in pre-clinical animal models. Previously, research on EV-B group vaccines was mainly focused on CV-B3 vaccine development. Because the HFMD pathogen spectrum has changed, and the threat from EV-B virus-associated severe diseases has gradually increased, it is necessary to develop multivalent HFMD vaccines. This study summarizes the clinical symptoms of diseases caused by EVs, such as HFMD, myocarditis and encephalitis, and the related EV vaccine development progress. In conclusion, developing multivalent EV vaccines should be strongly recommended to prevent HFMD, myocarditis, encephalitis and other severe diseases.

## INTRODUCTION

Enteroviruses (EVs) are a genus of positive-sense single-stranded RNA viruses associated with several human and mammalian diseases. The EV genus consists of 12 species: EV-A-H, EV-J and Rhinovirus A, B and C.^1^ EVs cause a wide range of diseases, including hand, foot and mouth disease (HFMD), upper respiratory tract infection, diarrhea, viral myocarditis, encephalitis and aseptic meningitis (Table 1). The most common EV in Europe and the United States (US) is the echovirus (E), which is the main pathogen of aseptic meningitis, while HFMD caused by the EV-A group is prevalent in the western Pacific region. The EV-A group is responsible for more than 90% of HFMD cases.^2^ EV-A71 and coxsackievirus (CV) CV-A16 from the EV-A group are the main pathogens responsible for worldwide HFMD outbreaks.^3, 4^ In addition to EV-A71 and CV-A16, several other EV-A may also cause HFMD. In recent years, CV-A6 and CV-A10 have gradually replaced EV-A71 and CV-A16 as the main pathogens of HFMD outbreaks. For example, CV-A6 was the pathogen responsible for HFMD outbreaks in Taiwan in 2010, Japan in 2011, Thailand in 2012 and mainland China in 2013,^5, 6, 7, 8^ whereas CV-A10 was responsible for the HFMD outbreaks in France in 2010 and Wuhan, China in 2013.^2, 9^ In addition, EV-B may lead to sporadic HFMD cases,^10^ among which CV-B3 and CV-B5 have been the predominant etiological agents. For example, CV-B3 was the main pathogen responsible for the 2012 HFMD epidemic in Shijiazhuang, China.^11^ EV-B infections usually lead to serious illnesses. In the United States, 20 000–40 000 cases of acute myocarditis are caused by CV-B3 each year. Among patients with acute myocarditis, 3.5–8.5 of every 100 000 people (9000–20 000 in total) may develop dilated chronic myocarditis.^12^ In fact, CV-B5 and E30 are the main causes of encephalitis and aseptic meningitis outbreaks.^13^

Because no effective treatments for HFMD and other diseases caused by EVs exist, vaccines have become the most effective solution in preventing EV-related diseases. In December 2015, two inactivated EV-A71 vaccines, which were the first HFMD vaccines, were approved in mainland China for preventing severe HFMD.^14, 15^ In addition, a CV-A16 monovalent vaccine and an EV-A71-CV-A16 bivalent vaccine showed good efficacy in HFMD prevention according to the preclinical study results.^16, 17^ Although research on CV-B3 vaccine has been conducted for years, no vaccine is available to protect children from viral myocarditis caused by CV-B3 infection.

Moreover, the results of epidemiological surveillance show that the EVs epidemic characteristics are changing. CV-A6 and CV-A10 are gradually replacing EV-A71 and CV-A16 as the major pathogens of HFMD, and the rising prevalence of CV-B3, CV-B5 and E30 is increasing the likelihood of consequent severe diseases to accur in infants.^18^ Therefore, multivalent EV vaccines should be researched and developed to prevent infants and children from being infected with severe diseases.

## EV-RELATED DISEASE EPIDEMICS

### EV-A group viruses

EV-A infections are the main causes of HFMD and herpangina (HA). It is reported that the EV-A group is responsible for more than 90% of HFMD cases,^2^ among which EV-A71 and CV-A16 are the main pathogens of HFMD outbreaks worldwide.^3, 4^ EV-A71 is responsible for most severe cases and deaths.^19^ CV-A6 and CV-A10 may also cause HFMD outbreaks, while other viruses in the EV-A group mainly cause HA and may also lead to sporadic HFMD cases.

Since the first EV-A71 strain was isolated in California in 1969, EV-A71-related HFMD outbreaks have occurred worldwide, particularly in the western Pacific region. For example, EV-A71 was the predominant pathogen responsible for HFMD outbreaks in Thailand in 2008, 2009 and 2011, with EV-A71 positive rates of 56%, 32% and 29%, respectively.^20^ During the period 2008–2012, EV-A71 ranked as the primary pathogen responsible for HFMD outbreaks in several cities of mainland China. A total of 10 714 237 survivors and 3046 deaths were reported from 2008 to June 2014 in mainland China, with a case fatality rate of 0.03%.^21^ In another study based on enhanced HFMD surveillance, the risk of severe-case fatality was 3.0%, with >90% of deaths associated with EV-A71.^22^

As another major HFMD pathogen, CV-A16, which co-circulates with EV-A71, also caused many HFMD outbreaks. A CV-A16-related HFMD outbreak was first reported in Toronto, Canada in 1957; subsequently, such outbreaks were also reported in Sydney, Australia in 1991, England and Wales in 1994, Taiwan during 2002–2003, Singapore during 2002, 2005 and 2007, Vietnam in 2005, India in 2009, and Thailand in 2010.^19, 20, 23, 24, 25, 26, 27, 28^ In mainland China, CV-A16 was the main pathogen responsible for HFMD epidemics. Although CV-A16 infection was considered only to cause mild symptoms, a few CV-A16 patients also developed aseptic meningitis and encephalitis.^29^

Research groups in Taiwan and Japan found that other viruses in the EV-A group might also cause both HA and HFMD.^5^ Before 2007, CV-A6, CV-A4 and CV-A10 were the main HA pathogens, whereas EV-A71 and CV-A16 were the main HFMD pathogens. However, CV-A6 and CV-A10 attracted more attention recently because they replaced EV-A71 and CV-A16 as the main causes of HFMD outbreaks in many countries.^7, 8^ CV-A2, CV-A4, CV-A5, CV-A6, CV-A8, CV-A10, CV-A12 and CV-A14 were also detected in HFMD clinical samples.^7, 19^

CV-A6 was responsible for the 2008 HFMD outbreak in Finland. Thereafter, CV-A6 was reported to cause HFMD outbreaks in Singapore in 2009, Taiwan during 2009–2010, Spain in 2011, Japan in 2011, Thailand in 2012, California in 2012 and Edinburg, UK in 2013.^5, 6, 30, 31, 32, 33, 34^ In many cities of mainland China, CV-A6 emerged as the major etiologic agent of HFMD, replacing EV-A71 and CV-A16. CV-A6-related HFMDs were characterized by atypical HFMD symptoms such as nail loss, with high incidences in adults and during the winter.^18^

Among the 141 samples taken during the 2010 France HFMD/HA outbreak, 134 were EV-A, with 39.9% CV-A10 and 28% CV-A6.^2^ Regarding the 2013 HFMD outbreak in the city of Wuhan, China, 463 viral strains were identified from 3208 HFMD samples, with 190 (41.0%) identified as CV-A10, 111 (21.2%) identified as EV-A71 and 52 (11.2%) identified as CV-A16.^9^ Therefore, after EV-A71 and CV-A16, CV-A6 and CV-A10 have emerged as the most significant HFMD pathogens.

### EV-B group viruses

The EV-B group has a high infection rate in humans. In 2007, CV-B1 became the most commonly identified EV serotype in the United States, accounting for 25% of all EV infections with known serotypes. Reportedly, the HFMD-causing EV-B viruses included CV-B1-5, E7, E13 and E30. In addition to mild HFMD cases, the CV-B subgroup was also responsible for severe diseases such as myocarditis and encephalitis.^35^

CV-B3 may cause severe diseases such as viral myocarditis, dilated chronic myocarditis and encephalitis in children, although most clinical symptoms of CV-B3-HFMD are mild. In the past, CV-B3 mainly caused sporadic HFMD cases, as was reported in Korea, Singapore, mainland China and Taiwan.^8, 11, 36, 37, 38, 39^ Recently, in mainland China, CV-B3-related HFMD has been on the rise. In fact, during the HFMD outbreak in the Chinese city of Shijiazhuang in 2012, CV-B3 (3.02%, 26/861) was the second most predominant non-EV-A71/CV-A16 enterovirus, behind only CV-A10.^11^ In the 2012 HFMD epidemic in Jiangsu, CV-B3 was the second major pathogen, behind CV-A16.^40^ Therefore, with the rise of HFMD cases caused by CV-B3 infection, there is greater risk for HFMD patients to develop myocarditis, dilated chronic myocarditis and other severe diseases.

CV-B5 was the major pathogen of encephalitis outbreaks in South Korea and mainland China. CV-B5 was ranked the No.2 pathogen of the 2005 (26.58%, 21/79) and 2009 (19.63%, 21/107) HFMD epidemics in Korea.^36, 37^ For the 2009 HFMD epidemic in Shandong China, CV-B5 was also the second major pathogen (12.7%, 14/110), with 11 of the 14 CV-B5 patients (78.6% (11/14)) developing neurological symptoms.^41^ The US Centers for Disease Control and Prevention statistics showed that CV-B group viruses (types 1–5) might cause more than 500 million infections each year.^42^ In addition to CV-B3, other EV-B viruses may also cause myocarditis and encephalitis. EV surveillance in Taiwan found that CV-B1 was the fourth predominant EV (3.4%) in 2008, with CV-B2 the fifth most common (7%) in 2006, CV-B4 the fifth most common in 2000 and 2001 (4.5% and 1.5%, respectively), the second most common in 2004 (14.9%), and the third most common in 2008 (12.0%). CV-B1 was reported to cause several viral myocarditis outbreaks.^43, 44^

The most commonly isolated EVs in Europe are E6, E7, E9, E11, E13 and E30. Over the past 35 years, US EV surveillance annual reports showed that E35, E6, E9, E11 and E30 were the four most prevalent EVs. Among them, E30 attracted the most attention because it caused several aseptic meningitis outbreaks.^13^ The prevalence of E30 among HFMD patients has also trended upward in recent years. In HFMD patients in Taiwan in 2008, E30 was the most predominant among all echoviruses.^39^ From HFMD samples in Shandong, China 2010, 35 non-EV-A71/CV-A16 EVs were isolated, including eight identified as E30,^45^ whereas E30 was also detected among patients with severe HFMD from that region in 2010–2011.^46^

### EV-C group viruses

CV-A21, CV-A24 and E96 from the EV-C group may cause HFMD. Among the 2,067 suspected HFMD cases in Guangzhou, China in 2011, CA21, CA24 and E96 each accounted for one case.^47^ CV-A21 was reported to cause acute respiratory infection in adults, while CV-A24 was the main pathogen of acute hemorrhagic conjunctivitis, aseptic meningitis, corneal endothelitis, and acute flaccid paralysis (AFP). E96 was also associated with AFP.

## EV INFECTION-RELATED CLINICAL MANIFESTATIONS

EVs may cause a wide spectrum of acute diseases with clinical manifestations ranging from non-specific febrile illness, HFMD, HA, mild upper respiratory tract infection, and self-limiting gastroenteritis to more severe ones such as myocarditis, hepatitis, and encephalitis. However, different subgroups of EVs are associated with different clinical manifestations. For example, EV-A was associated with HFMD/HA,^2^ EV-B with myocarditis/encephalitis, and CV-A24/E70 with hemorrhagic conjunctivitis.

### EV-A group viruses

Mild diseases caused by EV-A viruses include HFMD/HA, upper respiratory tract infection, and diarrhea, while severe diseases include encephalitis and other neurological diseases. Severe HFMD symptoms are usually caused by EV-A71, with encephalitis and pulmonary edema being the most common. Although CV-A16 infections usually lead to mild symptoms, severe and fatal HFMD cases related to CV-A16 have been reported.^29^ The clinical symptoms of CV-A6-related HFMD patients differed from those of patients with other HFMD types. CV-A6 HFMD patients usually have an atypical clinical rash, with nail loss being a prognostic sign. CV-A10 may cause HFMD as well as a number of severe diseases, such as aseptic meningitis.^48^

### EV-B group viruses

Among the illnesses caused by EV-B virus infections, the mild ones include HFMD, as well as respiratory and gastrointestinal symptoms, while the severe diseases include myocarditis, encephalitis and hepatitis. Each year, more than five million people are infected with CV-B1-5 virus in the United States. Up to 12% of EV-B-infected patients may have myocarditis, with CV-B3 being the main pathogen of viral myocarditis.^49^ Of considerable importance because myocarditis commonly goes undiagnosed, studies have shown that patients with CV-B3 infection were also at risk of myocarditis, even in the absence of HFMD symptoms. CV-B5 is also associated with encephalitis.^50^

## THE DEVELOPMENT OF EV VACCINES

Because EV-A71 and CV-A16 are the major HFMD pathogens, their vaccine development has progressed the fastest (Table 2). The EV-A71 vaccine has already shown substantial protective effect against EV-A71-HFMD and EV-A71-related diseases in clinical trials. Advances in developing a CV-A16 vaccine animal model and good preclinical results on a CV-A16 monovalent vaccine and an EV-A71/CV-A16 bivalent vaccine also provide auspicious prospects for the prevention of EV-A71- and CV-A16-related diseases.^16, 17^

### EV-A71 vaccines

EV-A71 vaccines include the whole virus-inactivated vaccine, virus-like particle (VLP) vaccine and peptide vaccine, among which the whole virus-inactivated vaccine has progressed the fastest. Inactivated EV-A71 vaccines developed by mainland China, Taiwan and Singapore used the C4, B4 and B3 genotype strains, respectively.^52^ Those virus strains were cultured in Vero cells or human diploid cells, before they were inactivated with formaldehyde. After purification, these inactivated viruses were mixed with aluminum salt adjuvants to obtain the final product. Preclinical research showed that these vaccines may induce animals to generate neutralizing antibodies (NTAbs) and exhibited good efficacy. The NTAb level was positively correlated with the protective effect. In 2010, the National Taiwan Institute of Health (NHRI) began the first EV-A71 clinical trial in adults. The subjects were inoculated with 5 μg or 10 μg of EV-A71 vaccine, and the results showed that the EV-A71 vaccine had good safety and immunogenicity in adults.^52^ Currently, Singapore Inviragen also completed a phase I clinical trial of their EV-A71 vaccine (0.3 μg and 3 μg dosages), whereas three mainland China manufacturers have completed phase III clinical trials on their EV-A71 vaccines. In those phase III clinical trials, a total of 32 000 infants were vaccinated with EV-A71 vaccines. The clinical results showed that the protection rate against HFMD was >90% and that the protection rate against other EV-A71-related diseases was >80%. In addition, the proposed threshold level of NTAbs should be between 1:16 and 1:32 for EV-A71 vaccine protection effectiveness. Further research on the batch-to-batch consistency of those three EV-A71 vaccines in China substantiated high immunogenicity consistency among all batches.^64^ The EV-A71 vaccine of Sinovac (Beijing, China) showed an efficacy rate of 95.1% (95% CI 63.6, 99.3) against EV-A71-associated HFMD during the extended follow-up and an overall efficacy rate of 94.7% (95% CI 87.8, 97.6) at two years, which indicated that the EV-A71 vaccine could provide a sustained high level of protection against EV-A71-associated HFMDs up to two years.^65^ The phase III clinical trial for the EV-A71 vaccine developed by the Chinese Academy of Medical Sciences (CAMS; Kunming, Yunnan Province, China) using human diploid cells showed that the sero-conversion rate was 95% during the 2-year follow-up and that the protection rate was 97.4% and 100% against EV-A71-HFMD at the 1st and 2nd year, respectively.^66^ Although the subgenotypes of the EV-A71 vaccine strains were different in mainland China, Taiwan and Singapore, serum samples obtained from children immunized against the C4 strain in mainland China or the B3 strain in Taiwan had good cross-neutralizing capacity against other EV-A71 subtypes. All studies have shown that children immunized with a single C4 strain vaccine gained cross-protection against other EV-A71 genotypes. Another study showed that polio vaccine inoculation in infants, simultaneously or successively along with EV-A71 vaccine inoculation, would not interfere with the EV-A71 vaccine immunization effect.^67, 68^ To ensure the accuracy and comparability for detecting NTAbs and antigen content in clinical trials, the National Institute for Food and Drug Control (NIFDC) established the first national standard on EV-A71 anti-serum and first national standard on EV-A71 antigen, and standardized the dosages of the EV-A71 vaccines from these three manufacturers.^69^ On the basis of the studies of national standards, the first international standard for anti-EV-A71 serum was established by the National Institute for Biological Standards and Control (NIBSC) and NIFDC recently.^70^ According to these results, two inactivated EV-A71 vaccines from CAMS and Sinovac Ltd were approved successively for preventing severe HFMD in mainland China in December 2015.

### CV-A16 vaccine

With the successful experience regarding the inactivated EV-A71 vaccine, the research on a CV-A16 vaccine has also been focused on an inactivated vaccine. Two CV-A16 strains (CV-A16-SZ05 and CV-A16-G08) were inactivated by Cai *et al* with β-propiolactone, cultured on Vero cells and mixed with aluminum adjuvant. Mice were inoculated with this vaccine and the results showed that it could induce CV-A16-specific antibodies and an interferon-r (IFN-r) cellular immune response. The anti-CV-A16 serum could neutralize both homologous and heterologous CV-A16 strains and a mouse-adapted CV-A16 strain (MAV). CV-A16 mouse anti-serum could partially neutralize the virus attack on neonatal mice, while CV-A16-vaccinated mice may be protected against CV-A16 MAV attacks.^71^ Qi An *et al* also prepared a CV-A16 vaccine by inactivating the virus with β-propiolactone, culturing the virus on Vero cells and formulating with aluminum salt. This vaccine also showed a good protective effect in the neonatal mouse model.^72^ Yang *et al* used a human diploid cell substrate in preparing inactivated CV-A16 vaccine. In the mouse and rhesus immune response research, this vaccine demonstrated a dose-dependent effect in inducing NTAb responses. In addition, the serum had good neutralizing effects against gene A and B subtypes of CV-A16, and this vaccine could induce IFN-r cellular immune responses.^73^

Recent studies have focused on CV-A16 VLP vaccine development and showed that VLP vaccines appear promising. A CV-A16 VLP vaccine was made from recombinant baculovirus-infected Sf9 cells. Serum from mice immunized with this vaccine could neutralize homologous (CV-A16/SZ05) and heterologous (CV-A16/GX08) strains of CV-A16 *in vitro*, and protect mice against attacks from lethal doses of CV-A16 virus challenge. Moreover, Zhao *et al*^61^ produced a CV-A16 VLP vaccine using *Saccharomyces cerevisiae* cells. This vaccine could also induce NTAbs and IgG antibodies, as well as cellular immune responses in mice. In the virus challenge test, this immune serum could protect neonatal mice against the lethal attack from a CV-A16 challenge.

### EV-A71 and CV-A16 bivalent vaccine

Because EV-A71 and CV-A16 are the major pathogens of HFMD and other severe diseases, monovalent EV-A71 vaccine and monovalent CV-A16 vaccine were successfully developed and have shown some successful pre-clinical progress. Several institutes have recently begun to develop EV-A71-CV-A16 bivalent vaccines.^62, 74^

#### Inactivated bivalent vaccine

Recent studies have proved the feasibility and effectiveness of inactivated bivalent vaccines. Cai *et al* evaluated the immunogenicity and protective efficacy of the bivalent EV-A71/CV-A16 vaccine prepared on Vero cell substrates in the mouse model. Similar to the monovalent vaccine, serum from mice immunized with bivalent vaccine showed the same capacity in neutralizing EV-A71 or CV-A16 virus *in vitro*. Bivalent vaccine may protect animals against both EV-A71 and CV-A16 viruses, whereas monovalent vaccine may only protect against one virus. No interference was observed between EV-A71 and CV-A16 immunization.^16^ Sun *et al*^74^ further demonstrated the protective effect of the inactivated bivalent EV-A71/CV-A16 vaccine in a mouse model *in vivo*.

#### VLP bivalent vaccine

Similar to inactivated monovalent vaccines, bivalent EV-A71/CV-A16 VLP vaccines may protect against both viruses. Gong *et al* used insect cells using baculovirus vectors to express EV-A71/CV-A16 VLPs. Immunogenicity was compared between monovalent EV-A71 VLPs, monovalent CV-A16 VLPs and bivalent VLPs vaccine. Serum from mice immunized with aluminum- or CpG-adjuvant VLPs bivalent vaccine could neutralize EV-A71 and CV-A16 *in vitro*.^62^ Ku *et al* also demonstrated that EV-A71/CV-A16-VLPs could induce high levels of antibodies, and protect mice from EV-A71 and CV-A16 infections.^17^ Sun *et al* investigated EV-A71/CV-A16 VLPs expressed on insect cells, and their research showed that the VLPs could protect neonatal mice from lethal EV-A71/CV-A16 challenge and demonstrated that NTAbs also had cross-protection against other sub-genotypes of EV-A71 and CV-A16.^74^ Liu *et al*^75^ recently found that CV-A10 VLPs efficiently induced antibodies capable of neutralizing CV-A10 infection *in vitro*.

### EV-B group virus vaccines

Some studies have reported on CV-B1-CV-B6 joint inactivated vaccines and vaccines for CV-B3 and CV-B1.^76, 77^ Because CV-B3 is the major cause of viral myocarditis and encephalitis, the study of EV-B enterovirus vaccines has been focused on CV-B3, particularly the attenuated CV-B3 vaccine. Virulence research on CV-B3 was mainly focused on the 5′-UTR region and the viral capsid protein region. The changes of some important nucleotide sites in the 5′-UTR region may affect virus binding with cardiomyocytes. In addition, the ribosome-binding sites of the 5′-UTR region (IRES) are the key sites for virus infection and translation.^78^ With mutations of those IRES sites, the mutant virus cannot cause myocarditis or myocardial necrosis in mice.^79^ With mutations of key VP1, VP2 and VP3 amino acids for CV-B3, the pathogenicity of the mutant virus also decreased significantly. However, the attenuated strain may still induce NTAbs in mice and may protect mice from lethal CV-B3 challenge. The decline in virulence after amino-acid mutations in the viral capsid protein may be due to the weak interaction between the virus and the receptor. More research is necessary to better understand the safety of the attenuated vaccine, notably the possible tissue damage caused by the attenuated virus as well as virulence recovery from the virus repair. Research on CV-B3 vaccine includes DNA vaccines, genetically engineered vaccines and the inactivated vaccine.^80^ All three vaccines reported above showed protective effects in mice against myocarditis caused by CV-B3. Moreover, the protein vaccine showed superior protection compared with the DNA vaccine.

## SUMMARY

EVs are among the most common disease-causing viruses in humans, particularly in infants and children. There is a wide spectrum of clinical manifestations caused by EV infections with varying degrees of severity. EV-A71 and CV-A16 were the main causes of HFMD epidemics in the western Pacific region. After 2008, CV-A6 and CV-A10 were the main pathogens of HFMD outbreaks. The prevalence of CV-A6 in mainland China and Southeast Asian countries after 2013 presented new challenges in HFMD prevention. Recently, the presence of CV-B3, CV-B5 and E30 among HFMD patients has trended upwards, which may lead to higher risk of more severe diseases for those patients.

Vaccine is the most effective and economical tool to control HFMD. In December 2015, two EV-A71 vaccines were approved for use in mainland China. The cost of immunization is ~$30 per dose with two doses required for primary immunization,^81^ whereas the total costs per patient for severe HFMD and mild HFMD were $2149.47 and $513.22, respectively. Furthermore, the loss of disability-adjusted life years of the two disease forms were, respectively 3.47 and 1.76 person-years per 1000 persons in rural central China, from 2011 to 2013, and the cost of illness for EV-A71-associated disease in China was estimated as >$450 million in 2013.^82^ Two reports forecasted that routine immunization with a 70% or 90% efficacious EV-A71 vaccine sold at $25 or $75 per dose, respectively, would be of great economic value.^83, 84^

The change in the HFMD pathogen spectrum and the threat of severe diseases caused by EV-B group viruses both indicate the necessity of developing a multivalent HFMD vaccine. Increasing numbers of investigators have recognized the importance of multivalent HFMD vaccines, which are mainly focused on EV-A71, CV-A16, CV-A6 and CV-A10.^75, 85, 86^ Recently, a trivalent-inactivated EV-A71/CV-A16/CV-A6 vaccine showed good protection from lethal challenge against each homologous virus in mice, which was similar to that of the corresponding monovalent vaccine groups.^87^ Moreover, a combination of formalin-inactivated EV-A71, CV-A6, CV-A10 and CV-A16 multivalent vaccine candidates could elicit serotype-specific neutralizing antibody responses in mice and rabbits, and no cross-neutralization efficacy was found among these viruses.^88^ However, many related studies are limited, particularly the national surveillance systems for non-EV-A71 and non-CV-A16 EVs in endemic areas. Critically important is the decision regarding which EV serotypes should be incorporated in the development of a multivalent vaccine. How should the appropriate strain of each EV serotype be chosen? How should the immunogenicity be evaluated and how should the protective efficacy be standardized? How should the immune interaction between these incorporated EV antigens be addressed? Could these vaccines induce an antibody-dependent enhancement effect? Are multiple vaccines economically feasible? Other questions remain.

To control HFMD and other severe diseases caused by EV infections in the pediatric population, the enhanced surveillance of related diseases caused by EV-A71 and CV-A16, as well as CV-A6, CV-A10, CV-B3, CV-B5 and E30 has been suggested. Concurrently, the development of a multivalent vaccine based on both EV-A71 and CV-A16 is urgently needed.

## Figures and Tables

**Table 1:** The clinical manifestation spectrum of enterovirus-associated diseases

**Enterovirus group**	**Mild disease**	**Severe disease**	**Comment**
EV-A	HFMD#^1^	NP-AFP#^4^	Associated with EV-A71#^1, 4, 5, 6, 7^; associated with CV-A16#^1, 2, 5, 6^; associated with CV-A6 or CV-A10^1, 2, 3^
	Herpangina#^2^	Aseptic meningitis#^5^	
	Onychomadesis#^3^	Encephalitis#^6^	
	Pharyngitis	Death#^7^	
	Diarrhea	Pulmonary edema	
	Respiratory symptoms	Neuromyelitis Optica	
	–	Infant sepsis	
EV-B	HFMD#^1^	NP-AFP#^4^	Associated with CV-B3#^1, 7, 8, 9^; associated with CV-B5 or E30#^1, 4, 5, 6, 7^
	Herpangina	Aseptic meningitis#^5^	
	Diarrhea	Encephalitis#^6^	
	Respiratory symptoms#^8^	Myocarditis#^9^	
	–	Death#^7^	
	–	Acute transverse myelitis	
	–	Acute pancreatitis	
	–	Papillitis	
	–	Hepatitis	
EV-C	HFMD#^1^	NP-AFP#^4^	Associated with CV-A22 or CV-A24#^1, 2, 5, 10^
	Herpangina#^2^	Aseptic meningitis#^5^	
	Acute hemorrhagic conjunctivitis#^10^	Hopkins syndrome	
	Corneal endothelial dysfunction	–	
Other EVs	HFMD#^1^	ND	Associated with rhinovirus#^1, 8^
	Respiratory symptoms#^8^	–	

Abbreviations: not determined, ND; non-polio acute flaccid paralysis, NP-AFP.

# Typical clinical manifestation.

**Table 2:** Research and development progresses regarding EV-A71 and CV-A16 vaccines

**EV vaccine**	**Vaccine type**	**Vaccine strain**	**Subgenotype**	**Cell substrate**	**R&D status**	**Organization (country/region)**
EV-A71 monovalent vaccine	Inactivated	FY-23	C4	KMB17	Production and registration approval	CAMS (China)^14^
	Inactivated	H07	C4	Vero	Production and registration approval	Sinovac (China)^15^
	Inactivated	FY7VP5	C4	Vero	Phase 3 clinical trial completed	Beijing Vigoo (China)^51^
	Inactivated	E59	B4	Vero	Phase 1 clinical trial completed	NHRI (Taiwan)^52^
	Inactivated	–	B3	Vero	Phase 1 clinical trial completed	Inviragen (Singapore)^53^
	Inactivated	E59	B4	Vero	Pre-clinical	Adimmune Corporation (Taiwan)^54^
	Attenuated live virus	BrCr	A	Vero	Pre-clinical	NIID (Japan)^55^
	VLP	neu	C2	Sf9	Pre-clinical	National Taiwan University (Taiwan)^56^
	VLP	G082	C4	Sf9	Pre-clinical	IPS-CAS (China)^57^
CV-A16 monovalent vaccine	Inactivated	SZ05	B1b	Vero	Pre-clinical	IPS-CAS (China)^58^
	Inactivated	CC024	B	Vero	Pre-clinical	First Hospital of Jilin University (China)^59^
	Peptide	SZ05	B1b	Synthetic	Pre-clinical	IPS-CAS (China)^60^
	VLP	GD09/119	B	Saccharomyces cerevisiae	Pre-clinical	Beijing Institute of Microbiology and Epidemiology (China)^61^
EV-A71/CV-A16 bivalent vaccine	Inactivated	FY573 & G08	C4 & B	Vero	Pre-clinical	IPS-CAS (China)^16^
	VLP	FY & 09-7	C4 & B1	Sf9	Pre-clinical	IB-CAS (China)^62^
	VLP	SB12736-SAR-03 & SB3512/SAR/00	–	Sf9	Pre-clinical	The University of Queensland (Australia)^63^
	VLP	G082 & SZ05	C4 & B1b	Sf9	Pre-clinical	HuaLan (China)^17^

Abbreviations: Chinese Academy of Medical Sciences, CAMS; Institute of Biophysics, Chinese Academy of Science, IB-CAS; Institute Pasteur of Shanghai, Chinese Academy of Sciences, IPS-CAS; National Health Research Institutes, NHRI; National Institute of Infectious Diseases, NIID; virus-like particle, VLP.

## References

[bib1] Fauquet CM, Mayo MA, Maniloff J et al. Virus Taxonomy, Eighth Report of the International Committee on Taxonomy of Viruses. Elsevier Academic Press: San Diego. 2005, 757–778.

[bib2] Mirand A, Henquell C, Archimbaud C et al. Outbreak of hand, foot and mouth disease/herpangina associated with coxsackievirus A6 and A10 infections in 2010, France: a large citywide, prospective observational study. Clin Microbiol Infect 2012; 18: E110–E118. 22404077 10.1111/j.1469-0691.2012.03789.x

[bib3] Mao Q, Wang Y, Yao X et al. Coxsackievirus A16: epidemiology, diagnosis, and vaccine. Hum Vaccin Immunother 2014; 10: 360–367. 24231751 10.4161/hv.27087 PMC4185891

[bib4] Yip CC, Lau SK, Woo PC et al. Human enterovirus 71 epidemics: what's next? Emerg Health Threats J 2013; 6: 19780. 24119538 10.3402/ehtj.v6i0.19780 PMC3772321

[bib5] Chen YJ, Chang SC, Tsao KC et al. Comparative genomic analysis of coxsackievirus A6 strains of different clinical disease entities. PLoS One 2012; 7: e52432. 23300669 10.1371/journal.pone.0052432 PMC3530459

[bib6] Fujimoto T, Iizuka S, Enomoto M et al. Hand, foot, and mouth disease caused by coxsackievirus A6, Japan, 2011. Emerg Infect Dis 2012; 18: 337–339. 22304983 10.3201/eid1802.111147 PMC3310456

[bib7] Puenpa J, Chieochansin T, Linsuwanon P. Hand, foot, and mouth disease caused by coxsackievirus A6, Thailand, 2012. Emerg Infect Dis 2013; 19: 641–643. 23631943 10.3201/eid1904.121666 PMC3647428

[bib8] Li JL, Yuan J, Yang F et al. Epidemic characteristics of hand, foot, and mouth disease in southern China, 2013: coxsackievirus A6 has emerged as the predominant causative agent. J Infect 2014; 69: 299–303. 24731881 10.1016/j.jinf.2014.04.001

[bib9] Yang Q, Ding J, Cao J et al. Epidemiological and etiological characteristics of hand, foot, and mouth disease in Wuhan, China from 2012 to 2013: outbreaks of coxsackieviruses A10. J Med Virol 2015; 87: 954–960. 25754274 10.1002/jmv.24151

[bib10] Ni H, Yi B, Yin J et al. Epidemiological and aetiological characteristics of hand, foot and mouth disease in Ningbo, China, 2008-2011. J Clin Virol 2012; 54: 342–348. 22652041 10.1016/j.jcv.2012.04.021

[bib11] Tian H, Zhang Y, Sun Q et al. Prevalence of multiple enteroviruses associated with hand, foot, and mouth disease in Shijiazhuang City, Hebei province, China: outbreaks of coxsackieviruses a10 and b3. PLoS One 2014; 9: e84233. 24392117 10.1371/journal.pone.0084233 PMC3879295

[bib12] Kim DS, Nam JH. Characterization of attenuated coxsackievirus B3 strains and prospects of their application as live-attenuated vaccines. Expert Opin Biol Ther 2010; 10: 179–190. 20088713 10.1517/14712590903379502

[bib13] Kim HJ, Kang B, Hwang S et al. Epidemics of viral meningitis caused by echovirus 6 and 30 in Korea in 2008. Virol J 2012; 9: 38. 22336050 10.1186/1743-422X-9-38 PMC3298778

[bib14] China Food and Drug AdministrationCFDA PIZHUN CHANGDAO BINGDU 71XING MIEHUOYIMIAO SHENGCHAN SHANGSHI. Chinese. CFDA: Beijing. 2015. Available at http://www.sfda.gov.cn/WS01/CL0051/136853.html (accessed on 27 January 2016).

[bib15] China Food and Drug AdministrationCFDA PIZHUN SHANGSHIYAOPIN GONGGAO. Chinese. CFDA: Beijing. 2016. Available at http://www.sda.gov.cn/WS01/CL0087/142000.html (accessed on 27 January 2016).

[bib16] Cai Y, Ku Z, Liu Q et al. A combination vaccine comprising of inactivated enterovirus 71 and coxsackievirus A16 elicits balanced protective immunity against both viruses. Vaccine 2014; 32: 2406–2412. 24657161 10.1016/j.vaccine.2014.03.012

[bib17] Ku Z, Liu Q, Ye X et al. A virus-like particle based bivalent vaccine confers dual protection against enterovirus 71 and coxsackievirus A16 infections in mice. Vaccine 2014; 32: 4296–4303. 24950363 10.1016/j.vaccine.2014.06.025

[bib18] Bian L, Wang Y, Yao X et al. Coxsackievirus A6: a new emerging pathogen causing hand, foot and mouth disease outbreaks worldwide. Expert Rev Anti Infect Ther 2015; 13: 1061–1071. 26112307 10.1586/14787210.2015.1058156

[bib19] Ang LW, Koh BK, Chan KP et al. Epidemiology and control of hand, foot and mouth disease in Singapore, 2001-2007. Ann Acad Med Singapore 2009; 28: 106–112. 19271036

[bib20] Linsuwanon P, Puenpa J, Huang SW et al. Epidemiology and seroepidemiology of human enterovirus 71 among Thai populations. J Biomed Sci 2014; 21: 16. 24548776 10.1186/1423-0127-21-16 PMC3937078

[bib21] Liu SL, Pan H, Liu P et al. Comparative epidemiology and virology of fatal and nonfatal cases of hand, foot and mouth disease in mainland China from 2008 to 2014. Rev Med Virol 2015; 25: 115–128. 25704797 10.1002/rmv.1827

[bib22] Xing W, Liao Q, Viboud C et al. Hand, foot and mouth disease in China, 2008-12: an epidemiological study. Lancet Infect Dis 2014; 14: 308–318. 24485991 10.1016/S1473-3099(13)70342-6 PMC4035015

[bib23] Robinson CR, Doane FW, Rhodes AJ. Report of an outbreak of febrile illness with pharyngeal lesions and exanthem: Toronto, summer 1957; isolation of group A Coxsackie virus. Can Med Assoc J 1958; 79: 615–621. 13585281 PMC1830188

[bib24] Ferson MJ, Bell SM. Outbreak of coxsackievirus A16 hand, foot, and mouth disease in a child day-care center. Am J Public Health 1991; 81: 1675–1676. 1746672 10.2105/ajph.81.12.1675 PMC1405294

[bib25] Bendig JW, Fleming DM. Epidemiological, virological, and clinical features of an epidemic of hand, foot, and mouth disease in England and Wales. Commun Dis Rep CDR Rev 1996; 6: R81–R86. 8664928

[bib26] Chang LY. Enterovirus 71 in Taiwan. Pediatr Neonatol 2008; 49: 103–112. 19054914 10.1016/S1875-9572(08)60023-6

[bib27] Tu PV, Thao NTT, Perera D et al. Epidemiologic and virologic investigation of hand, foot and mouth disease, Southern Vietnam, 2005. Emerg Infect Dis 2007; 13: 1733–1741. 18217559 10.3201/eid1311.070632 PMC3375788

[bib28] Kar BR, Dwibedi B, Kar SK. An outbreak of hand, foot and mouth disease in Bhubaneswar, Odisha. Indian Pediatr 2013; 50: 139–142. 22728634 10.1007/s13312-013-0033-0

[bib29] Wang CY, Li Lu F, Wu MH et al. Fatal coxsackievirus A16 infection. Pediatr Infect Dis J 2004; 23: 275–276. 15014311 10.1097/01.inf.0000115950.63906.78

[bib30] Wu Y, Yeo A, Phoon MC et al. The largest outbreak of hand, foot and mouth disease in Singapore in 2008: the role of enterovirus71 and coxsackievirus A strains. Int J Infect Dis 2010; 14: e1076. 20952237 10.1016/j.ijid.2010.07.006

[bib31] Montes M, Artieda J, Piñeiro LD et al. Hand, foot, and mouth disease outbreak and coxsackievirus A6, northern Spain, 2011. Emerg Infect Dis 2013; 19: 676–678. 10.3201/eid1904.121589 PMC3647425 23751014

[bib32] Puenpa J, Mauleekoonphairoj J, Linsuwanon P et al. Prevalence and characterization of enterovirus infections among pediatric patients with hand foot mouth disease, herpangina and influenza like illness in Thailand, 2012. PLoS One 2014; 9: e98888. 24887237 10.1371/journal.pone.0098888 PMC4041783

[bib33] Centers for Disease Control and Prevention (CDC). Notes from the field: severe hand, foot, and mouth disease associated with coxsackievirus A6–Alabama, Connecticut, California, and Nevada, November 2011-February 2012. MMWR Morb Mortal Wkly Rep 2012; 61: 213–214. 22456122

[bib34] Sinclair C, Gaunt E, Simmonds P et al. Atypical hand, foot, and mouth disease associated with coxsackievirus A6 infection, Edinburgh, United Kingdom, January to February 2014. Euro Surveill 2014; 19: 20745. 24698138 10.2807/1560-7917.es2014.19.12.20745

[bib35] Kim DS, Nam JH. Application of attenuated coxsackievirus B3 as a viral vector system for vaccines and gene therapy. Hum Vaccin 2011; 7: 410–416. 21389776 10.4161/hv.7.4.14422

[bib36] Baek K, Park K, Jung E et al. Molecular and epidemiological characterization of enteroviruses isolated in Chungnam, Korea from 2005 to 2006. J Microbiol Biotechnol 2009; 19: 1055–1064. 19809266 10.4014/jmb.0810.584

[bib37] Baek K, Yeo S, Lee B et al. Epidemics of enterovirus infection in Chungnam Korea, 2008 and 2009. Virol J 2011; 8: 297. 21668960 10.1186/1743-422X-8-297 PMC3130694

[bib38] Shah VA, Chong CY, Chan KP et al. Clinical characteristics of an outbreak of hand, foot and mouth disease in Singapore. Ann Acad Med Singapore 2003; 32: 381–387. 12854382

[bib39] Hsu CH, Lu CY, Shao PL et al. Epidemiologic and clinical features of non-polio enteroviral infections in northern Taiwan in 2008. J Microbiol Immunol Infect 2011; 44: 265–273. 21524954 10.1016/j.jmii.2011.01.029

[bib40] Yao X, Bian LL, Lu WW et al. Enterovirus spectrum from the active surveillance of hand foot and mouth disease patients under the clinical trial of inactivated Enterovirus A71 vaccine in Jiangsu, China, 2012-2013. J Med Virol 2015; 87: 2009–2017. 26010334 10.1002/jmv.24275

[bib41] Chen P, Tao Z, Song Y et al. A coxsackievirus B5-associated aseptic meningitis outbreak in Shandong Province, China in 2009. J Med Virol 2013; 85: 483–489. 23212939 10.1002/jmv.23478

[bib42] Moore M, Kaplan MH, McPhee J et al. Epidemiologic, clinical, and laboratory features of Coxsackie B1-B5 infections in the United States, 1970-79. Public Health Rep 1984; 99: 515–522. 6091168 PMC1424625

[bib43] Bae EY, Lee EJ, Han SB et al. A case of myocarditis following neonatal meningitis caused by coxsackievirus B1 in spite of intravenous immunoglobulin treatment. J Trop Pediatr 2014; 60: 164–167. 24122863 10.1093/tropej/fmt086

[bib44] Tapparel C, Siegrist F, Petty TJ et al. Picornavirus and enterovirus diversity with associated human diseases. Infect Genet Evol 2013; 14: 282–293. 23201849 10.1016/j.meegid.2012.10.016

[bib45] Pei YW, Wang JX, Lin Y et al. Genetic analysis of Echovirus 30 isolated from hand-foot-mouth disease causes and healthy controls from Shandong, China in 2010. Chin J Viral Dis 2013; 3: 367–371.

[bib46] Zhang T, Du J, Xue Y et al. Epidemics and frequent recombination within species in outbreaks of human enterovirus B-associated hand,foot and mouth disease in Shandong China in 2010 and 2011. PLoS One 2013; 8: e67157. 23840610 10.1371/journal.pone.0067157 PMC3686723

[bib47] He P, Chen C, Di B et al. Spectrum analysis of hand, foot and mouth disease in Guangzhou in 2011. J Trop Med 2012; 12: 613–616.

[bib48] Kumar A, Shukla D, Kumar R et al. Molecular identification of enteroviruses associated with aseptic meningitis in children from India. Arch Virol 2013; 158: 211–215. 22975986 10.1007/s00705-012-1476-7

[bib49] Martino TA, Liu P, Sole MJ. Viral infection and the pathogenesis of dilated cardiomyopathy. Circ Res 1994; 74: 182–188. 8293557 10.1161/01.res.74.2.182

[bib50] Dumaidi K, Frantzidou F, Papa A et al. Enterovirus meningitis in Greece from 2003-2005: diagnosis, CSF laboratory findings, and clinical manifestations. J Clin Lab Anal 2006; 20: 177–183. 16960900 10.1002/jcla.20129 PMC6807394

[bib51] Zhu FC, Meng FY, Li JX et al. Efficacy, safety, and immunology of an inactivated alum-adjuvant enterovirus 71 vaccine in children in China: a multicentre, randomised, double-blind, placebo-controlled, phase 3 trial. Lancet 2013; 381: 2024–2032. 23726161 10.1016/S0140-6736(13)61049-1

[bib52] Cheng A, Fung CP, Liu CC et al. A phase I, randomized, open-label study to evaluate the safety and immunogenicity of an enterovirus 71 vaccine. Vaccine 2013; 31: 2471–2476. 23541623 10.1016/j.vaccine.2013.03.015

[bib53] National Institutes of Health Safety and Immunogenicity Study of an Inactivated Vaccine Against Hand, Foot and Mouth Disease Caused by Enterovirus 71. ClinicalTrials.gov Identifier: NCT01376479. NIH: USA. 2015. Available at https://prsinfo.clinicaltrial.gov/ct2/show/nct01376479?term=inviragen±%28singapore%29±pte±ltd.&rank=1%202013 (accessed 27 January 27 2016).

[bib54] Wu CY, Lin YW, Kuo CH et al. Inactivated enterovirus 71 vaccine produced by 200-L scale serum-free microcarrier bioreactor system provides cross-protective efficacy in human SCARB2 transgenic mouse. PLoS One 2015; 10: e0136420. 26287531 10.1371/journal.pone.0136420 PMC4543551

[bib55] Arita M, Nagata N, Iwata N et al. An attenuated strain of enterovirus 71 belonging to genotype A showed a broad spectrum of antigenicity with attenuated neurovirulence in cynomolgus monkeys. J. Virol 2007; 81: 9386–9395. 17567701 10.1128/JVI.02856-06 PMC1951441

[bib56] Chung YC, Ho MS, Wu JC et al. Immunization with virus-like particles of enterovirus 71 elicits potent immune responses and protects mice against lethal challenge. Vaccine 2008; 26: 1855–1862. 18329759 10.1016/j.vaccine.2008.01.058

[bib57] Ku Z, Ye X, Huang X et al. Neutralizing antibodies induced by recombinant virus-like particles of enterovirus 71 genotype C4 inhibit infection at pre- and post-attachment steps. PLoS One 2013; 8: e57601. 23451250 10.1371/journal.pone.0057601 PMC3579802

[bib58] Liu Q, Shi J, Huang X et al. A murine model of coxsackievirus A16 infection for anti-viral evaluation. Antiviral Res 2014; 105: 26–31. 24583030 10.1016/j.antiviral.2014.02.015

[bib59] Li J, Liu G, Liu X et al. Optimization and characterization of candidate strain for coxsackievirus A16 inactivated vaccine. Viruses 2015; 7: 3891–3909. 26193302 10.3390/v7072803 PMC4517132

[bib60] Shi J, Huang X, Liu Q et al. Identification of conserved neutralizing linear epitopes within the VP1 protein of coxsackievirus A16. Vaccine 2013; 31: 2130–2136. 23499595 10.1016/j.vaccine.2013.02.051

[bib61] Zhao H, Li HY, Han JF et al. Virus-like particles produced in *Saccharomyces cerevisiae* elicit protective immunity against coxsackievirus A16 in mice. Appl Microbiol Biotechnol 2013; 97: 10445–10452. 24085395 10.1007/s00253-013-5257-3

[bib62] Gong M, Zhu H, Zhou J et al. Cryo-electron microscopy study of insect cell-expressed enterovirus 71 and coxsackievirus a16 virus-like particles provides a structural basis for vaccine development. J Virol 2014; 88: 6444–6452. 24672036 10.1128/JVI.00200-14 PMC4093858

[bib63] Somasundaram B, Chang C, Fan YY et al. Characterizing enterovirus 71 and coxsackievirus A16 virus-like particles production in insect cells. Methods 2016; 95: 38–45. 26410190 10.1016/j.ymeth.2015.09.023

[bib64] Chen YJ, Meng FY, Mao Q et al. Clinical evaluation for batch consistency of an inactivated enterovirus 71 vaccine in a large-scale phase 3 clinicaltrial. Hum Vaccin Immunother 2014; 10: 1366–1372. 24633366 10.4161/hv.28397 PMC4896521

[bib65] Li JX, Song YF, Wang L et al. Two-year efficacy and immunogenicity of Sinovac enterovirus 71 vaccine against hand, foot and mouth disease in children. Expert Rev Vaccines 2015; 15: 129–137. 26460695 10.1586/14760584.2016.1096782

[bib66] Liu L, Mo Z, Liang Z et al. Immunity and clinical efficacy of an inactivated enterovirus 71 vaccine in healthy Chinese children: a report of further observations. BMC Med 2015; 13: 226. 26381232 10.1186/s12916-015-0448-7 PMC4574357

[bib67] Zhu FC, Liang ZL, Li XL et al. Immunogenicity and safety of an enterovirus 71 vaccine in healthy Chinese children and infants: a randomised, double-blind, placebo-controlled phase 2 clinical trial. Lancet 2013; 381: 1037–1045. 23352749 10.1016/S0140-6736(12)61764-4

[bib68] Mao Q, Wang Y, Shao J et al. The compatibility of inactivated-enterovirus 71 vaccination with coxsackievirus A16 and Poliovirus immunizations in humans and animals. Hum Vaccin Immunother 2015; 11: 2723–2733. 25715318 10.1080/21645515.2015.1011975 PMC4685687

[bib69] Liang Z, Mao Q, Gao Q et al. Establishing China's national standards of antigen content and neutralizing antibody responses for evaluation of enterovirus 71 (EV71) vaccines. Vaccine 2011; 29: 9668–9674. 22015395 10.1016/j.vaccine.2011.10.018

[bib70] Liang Z, Wang J. EV71 vaccine, an invaluable gift for children. Clin Transl Immunology 2014; 3: e28. 25505956 10.1038/cti.2014.24 PMC4237031

[bib71] Cai Y, Liu Q, Huang X et al. Active immunization with a coxsackievirus A16 experimental inactivated vaccine induces neutralizing antibodies and protects mice against lethal infection. Vaccine 2013; 31: 2215–2221. 23499596 10.1016/j.vaccine.2013.03.007

[bib72] Qi An W, Guo Su Z, Wen Pan R et al. The immunogenicity and protection effect of the BPL-inactivated CA16 vaccine in different animal systems. Hum Vaccin Immunother 2014; 10: 628–639. 24401488 10.4161/hv.27295 PMC4130287

[bib73] Yang E, Cheng C, Zhang Y et al. Comparative study of the immunogenicity in mice and monkeys of an inactivated CA16 vaccine made from a human diploid cell line. Hum Vaccin Immunother 2014; 10: 1266–1273. 24583556 10.4161/hv.28083 PMC4896597

[bib74] Sun S, Jiang L, Liang Z et al. Evaluation of monovalent and bivalent vaccines against lethal enterovirus 71 and coxsackievirus A16 infection in newborn mice. Hum Vaccin Immunother 2014; 10: 2885–2895. 25483672 10.4161/hv.29823 PMC5443096

[bib75] Liu Q, Tong X, Huang Z. Towards broadly protective polyvalent vaccines against hand, foot and mouth disease. Microbes Infect 2015; 17: 155–162. 25449959 10.1016/j.micinf.2014.11.004

[bib76] See DM, Tilles JG. Occurrence of coxsackievirus hepatitis in baby rabbits and protection by a formalin-inactivated polyvalent vaccine. Proc Soc Exp Biol Med 1997; 216: 52–56. 9316610 10.3181/00379727-216-44155

[bib77] Wu XY, Zhao TQ, Tian Y et al. A bivalent VP1 gene vaccine against Coxsackie virus B1/B3. Natl Med J China 2001; 81: 480–484. 11798923

[bib78] Liu Z, Carthy CM, Cheung P et al. Structural and functional analysis of the 5′ untranslated region of coxsackievirus B3 RNA: *In vivo* translational and infectivity studies of full-length mutants. Virology 1999; 265: 206–217. 10600593 10.1006/viro.1999.0048

[bib79] M'hadheb-Gharbi MB, El Hiar R, Paulous S et al. Role of GNRA motif mutations within stem-loop V of internal ribosome entry segment in coxsackievirus B3 molecular attenuation. J Mol Microbiol Biotechnol 2008; 14: 147–156. 17693702 10.1159/000107369

[bib80] Lan J, Gao Z, Xiong H et al. Generation of protective immune responses against coxsackievirus B3 challenge by DNA prime-protein boost vaccination. Vaccine 2011; 29: 6894–6902. 21803097 10.1016/j.vaccine.2011.07.049

[bib81] Economic Daily. EV71 SHOUZUKOU YIMIAO KAISHI JIEZHONG, MEIJI JIAGE BU CHAO 218 YUAN. Chinese. Beijing: ED, 2016. Available at http://baby.ce.cn/qt/201603/29/t20160329_3652260.shtml (accessed on 13 April 2016).

[bib82] Gan ZK, Jin H, Li JX et al. Disease burden of enterovirus 71 in rural central China: a community-based survey. Hum Vaccin Immunother 2015; 11: 2400–2405. 26158689 10.1080/21645515.2015.1059980 PMC4635911

[bib83] Lee BY, Wateska AR, Bailey RR et al. Forecasting the economic value of an enterovirus 71 (EV71) vaccine. Vaccine 2010; 28: 7731–7736. 20923711 10.1016/j.vaccine.2010.09.065 PMC2989421

[bib84] Li L, Yin H, An Z et al. Considerations for developing an immunization strategy with enterovirus 71 vaccine. Vaccine 2015; 33: 1107–1112. 25444807 10.1016/j.vaccine.2014.10.081

[bib85] Klein M, Chong P. Is a multivalent hand, foot, and mouth disease vaccine feasible? Hum Vaccin Immunother 2015; 11: 2688–2704. 26009802 10.1080/21645515.2015.1049780 PMC4685682

[bib86] Liu CC, Chow YH, Chong P et al. Prospect and challenges for the development of multivalent vaccines against hand, foot and mouth diseases. Vaccine 2014; 32: 6177–6182. 25218294 10.1016/j.vaccine.2014.08.064

[bib87] Caine EA, Fuchs J, Das SC et al. Efficacy of a trivalent hand, foot, and mouth disease vaccine against enterovirus 71 and coxsackieviruses A16 and A6 in mice. Viruses 2015; 7: 5919–5932. 26593938 10.3390/v7112916 PMC4664989

[bib88] Liu CC, Guo MS, Wu SR et al. Immunological and biochemical characterizations of coxsackievirus A6 and A10 viral particles. Antiviral Res 2016; 129: 58–66. 26899790 10.1016/j.antiviral.2016.02.008

